# Crystallized Gold

**Published:** 1856-01

**Authors:** 


### ARTICLE XIII.

### *Crystallized Gold.*

When Dr. Watts first attempted to prepare a form of sponge or crystalline gold for filling teeth, he experienced great difficulty from his ignorance of the details of dentistry. If he had been thoroughly acquainted with the modes of manipulation by which unexceptionable gold plugs were produced in the mouth, and with the varied influences by which their integrity may be subsequently endangered or destroyed, the profession and himself would have been saved a great deal of time, trouble, expense and mortification. Being, however, professionally and practically a chemist, he was obliged to appeal to dentists for their judgment and counsel. With this view he submitted a form of prepared gold to a few of the best operators with whom he was at that time acquainted, and upon whose judgment he thought he could most safely rely.

To these persons it was a new and extraordinary thing. Its singular qualities—the peculiar mode of using it—and the results attained with it, were equally remarkable. They were "astonished," "delighted;" in the heat of enthusiasm, reported it, "the greatest discovery of the age," and in letters to the Dr., it was lauded without limit, and set down as already a "perfect" thing. It was further said, that slight experience would enable comparatively unskillful persons to accomplish better work with it than could be produced with foil in the hands of operators of tried skill and large experience; that its use required less time than foil, while it could hardly be said to require *skill* at all. All this occurred before the gold had been offered for sale, or any directions published for its use.

The truth was, that crystal gold at this period should have been subjected to rigid critical trials and tests, particularly the "test of time," that Dr. W. might have had an opportunity to remedy its defects and perfect its character; but, he was unfortunately led to believe his first article to be all that the profession desired, and the prepared gold was therefore sent out to the world, accompanied by "directions" entirely authorized by the honest but incautious judgment so far rendered upon it. The profession were therein told that crystal gold required for its successful use, comparatively little time and skill, and that no specific instruments were necessary—the end of a broken file "working admirably !"

This was a great blunder—more—it was a *disaster!* It not only justified, but directed carelessness, and gave an entirely mistaken idea of the requirements and qualities of the gold. If a dentist, with some clumsy tool, perhaps the "broken file" aforesaid, filled a large cavity, half prepared, and the plug, less than half condensed, subsequently came out or went to pieces, he had followed "directions," and charged the failure to the "gold."—The "directions" were at fault, sadly, entirely, yet in preparing them, the Dr. had been misled, as just stated.

As might be supposed, or, at least, as we can now plainly see, crystal gold sent out in this way, met with a singular reception. The too sanguine operator, expecting too much with inadequate labor—with instruments, at best inappropriate, and most likely none at all to the purpose—released from any obligation to practice proper skill and care, essayed to fill teeth with sponge gold, and at once encountered difficulties. The Dr. was straightway inundated with letters from all directions ; the general idea pervading them seemed to be, that the writers had discovered valuable qualities in the new material, which they were anxious to make available to the fullest extent, and each would suggest some specific change, which would improve it for *his* purpose. One wanted it harder—another softer—with one, it crumbled, was too adhesive, or not adhesive enough, and so on; and here the Dr. committed the second great error.

Being so perfectly conversant with the laws regulating the crystallization of gold, in the large field opened up by his discoveries, as to be able, at pleasure, to vary the qualities and characteristics of the new material, and only anxious to learn what precise form of production the profession *wanted*, he actually undertook to manufacture varieties differing sufficiently in character to please this whole mass of correspondents; hoping, thereby, to hit upon the *single* form which would please them *all.* A great amount of time and money was wasted in this effort, and herein is the real cause of the great number of forms and qualities of prepared gold which have been manufactured here and sent over the country; their number and variety being distracting to the Dr. as well as the profession, and giving just cause for what came to be the general belief, that this diversity arose from some difficulty in the process of manufacture, and that uniformity was really unattainable, *which neither is, nor has, at any time, been true.*

The great confusion produced in the laboratory by attempting so many changes and modifications, necessarily interfered with the regular details of the manufacture, and hence it was, that impurities occasionally escaped detection. This accounts for the discolorations that have been noticed, a result deplorable in the extreme, but now most certainly and reliably guarded against.

This defect was, as we state, purely accidental; the process of manufacture involves no sort of necessity or excuse for the slightest impurity, and our determination to manufacture but the one variety, affords an additional security from such hazard.

In the course of this general effort to please everybody, a series of dark-brown, or wine-colored formations were produced, which possessed peculiar properties. Although not so yielding or plastic in the mass as the brighter forms, they were greatly admired for their *adhesiveness*, and had at once many friends. The general report coming to us, was, that the brown varieties wasted more or less, but readily adhered on pressure, while the bright varieties did not waste at all, and did not adhere so well.

Experience has since demonstrated the following facts in reference to these two varieties of prepared gold:

The bright forms, upon comparatively slight pressure, condense *solid.* If the face of the instrument used is *smooth*, though *uneven*, the added piece does not adhere, but comes up, taking a *perfect impression* of the portion already condensed;

9*

so perfect is its plasticity, and so fine its structure, that it will take a perfect impression of the most delicate etching, on steel or copper-plate, and completely render every inequality visible through a microscope of high power. But this very quality, invaluable, and *perfect* in this material, requires that it should be continually worked with *sharply and finely cut surfaces*, that the bearing surface of instruments should be always *kept* sharp, in order that the working surface of the plug may remain sufficiently rough for the new gold to obtain a hold upon it. The *chief* almost *sole* requirement of this gold, is *proper tools;* and the profession, as a mass, had anything but proper tools.

On the other hand, the brown varieties were more deceptive in their character, and by reason of their greater adhesiveness. The operator could, with the imperfect instruments then in use, and with a certain amount of pressure, produce plugs which were very hard, and to all appearance solid; but which, when examined by the aid of the microscope, were found to be not solid but porous.

It was into the (to the eye) invisible, but innumerable interstices of the apparently condensed mass, that the added gold insinuated itself. Though packed with comparatively smooth instruments, it would still, in this way, adhere finely; and yet this quality of adhesion was due to an imperfection. Operations of this character failed, would crumble to pieces and fall out, authorizing the statements about brick-dust, &c. A greater amount of pressure would make a plug more lasting in its character, but still leave it sufficiently porous to admit of the absorption of moisture. Still greater pressure would obviate the danger from the absorption of fluids, and yet leave the plug lighter than perfectly solid gold. And though perfectly solid plugs have been, and can at any time be made with it, such result required very perfect instruments, great labor, time and patience.

Nine-tenths of all the complaints which have been made against crystal gold—well-founded, ill-founded and unfounded—except, perhaps, the more or less slight tendency to crumbling, and, therefore, waste, which distinguished all the earlier productions, are due to these brown varieties, aided by the want of proper instructions and appropriate and well made instruments.

From all this, it became evident that crystal gold had pressing need of tools made for and adapted to itself. We at once determined to publish a popular treatise on the qualities and use of the gold, embodying all the available experience of skillful and successful operators, in regard to instruments. Dr. Dwinelle, of Cazenovia, N. Y., who had great experience in the use of all forms of crystal gold, and who was, with unvarying certainty and success, accomplishing with it the most astonishing triumphs, prepared this "Treatise" for us in an able and satisfactory manner, its sole defect consisting in the inability to distinctly represent, by means of plates, the most important peculiarities of the instruments.—We furthermore resolved to modify the character of the dark varieties, gradually approaching, as nearly as possible, the brighter forms, and, so soon as the "Treatise" and right instruments could be prepared, to cease their manufacture altogether. Accordingly, since about the first of July last, none of the brown varieties have been made.

Now, although in the hands of the same operator, *with right instruments*, there could be no just comparison, in all essential characteristics, between what we have termed the brown and bright varieties, yet we feared to let this *best* material go at once out among the profession, to be manipulated and tested with the instruments in general use, although we had, perhaps, from being originally misled, as before stated, contributed more than all others, to introduce and justify the use of poor tools. We, therefore, refused the repeated request of Dr. Dwinelle, and others, (that we would send it out,) solely on the grounds stated, that some general knowledge of the characteristics of crystal gold, and some specific knowledge of proper instruments should precede its general introduction. Our course, in this respect, has given offence to some persons, and we *may* have been wrong, but when the experienced dentists, with the *best* of instruments, shall try his hand upon the gold *now* sent out from this establishment, we think he will endorse our course, and thank us for it.

The "Treatise" and the efforts of manufacturers of dental instruments, to whom we are under obligations, have so far removed this difficulty, however, that this "reserved" gold is now *only* manufactured.

This highly "improved," and as we regard it, "*perfected*" article, is claimed to be free from the objections which have been urged against any previous form of sponge or crystal gold. It is soft, silky, not friable, condenses readily, makes a perfectly solid plug, with less pressure than the other varieties, and will not change in color, permanence, or other characteristics, in or out of the mouth, in the least. We are entirely aware of the great breadth of this statement, but we are willing to put every thing at hazard on its absolute truthfulnes. It *must*, (we have explained why,) be used with finely and sharply cut points, and be thoroughly condensed, yet it requires less pressure, to secure perfect consolidation, than any other form of gold now used.

We know that the varying character of crystalline gold, as heretofore made, its often real defects, and the difficulty, in most cases impossibility, of obtaining fit instruments, have prejudiced and weaned many from its use altogether. Yet the universal response to us proves that few such as have failed to discover qualities which would render it invaluable in their hands if not linked to imperfections in the material and uncertainty in the manufacture. To all such, and the entire dental profession, we say that the period of trial, experiment, change and uncertainty has passed. We make and abide by the merits of but one form of gold—uniform in all respects, except in the differing density which distinguishes the various numbers—susceptible of every use, and competent to every triumph which the most ardent admirer has ever hoped to accomplish with it.

A. J. Watts & Co.

Utica, N. Y., October 27th, 1855.

All persons having prepared gold, manufactured by us previous to the first of July last, are requested to forward the same to us, and exchange it for the perfected article.

A. J. W. & Co.

